# Simplified ChIP-exo assays

**DOI:** 10.1038/s41467-018-05265-7

**Published:** 2018-07-20

**Authors:** Matthew J. Rossi, William K. M. Lai, B. Franklin Pugh

**Affiliations:** Center for Eukaryotic Gene Regulation, Department of Biochemistry and Molecular Biology, The Pennsylvania State University, University Park, PA 16802 USA

## Abstract

ChIP-seq and ChIP-exo identify where proteins bind along any genome in vivo. Although ChIP-seq is widely adopted in academic research, it has inherently high noise. In contrast, ChIP-exo has relatively low noise and achieves near-base pair resolution. Consequently, and unlike other genomic assays, ChIP-exo provides structural information on genome-wide binding proteins. Construction of ChIP-exo libraries is technically difficult. Here we describe greatly simplified ChIP-exo methods, each with use-specific advantages. This is achieved through assay optimization and use of Tn5 tagmentation and/or single-stranded DNA ligation. Greater library yields, lower processing time, and lower costs are achieved. In comparing assays, we reveal substantial limitations in other ChIP-based assays. Importantly, the new ChIP-exo assays allow high-resolution detection of some protein-DNA interactions in organs and in as few as 27,000 cells. It is suitable for high-throughput parallelization. The simplicity of ChIP-exo now makes it a highly appropriate substitute for ChIP-seq, and for broader adoption.

## Abstract (web summary)

While ChIP-exo is low noise and highly informative regarding genome-wide binding proteins, libraries are difficult to construct. Here the authors present a simplified ChIP-exo method for high-resolution detection of interactions.

## Introduction

Chromatin immunoprecipitation (ChIP) is a long-standing method for detecting protein-DNA interactions in vivo^1,2^. Formaldehyde is used to covalently trap proteins at their in vivo binding locations. After quenching, chromatin is isolated and fragmented. Next, a protein of interest is immunoprecipitated and its attached DNA identified by either PCR, microarrays^3^, or deep sequencing (ChIP-seq)^4,5^; listed in order of increased genome coverage and resolution. ChIP-exo was developed as a variation of ChIP-seq to improve sensitivity and increase positional resolution by up to two orders of magnitude. It uses lambda exonuclease to digest sonicated chromatin to the formaldehyde-induced protein-DNA cross-linking point^6^. By providing near base pair (bp) resolution of protein-DNA interactions, structural insights into protein complex organization are gained. Version 1.0 of the ChIP-exo method was introduced for the SOLiD sequencing platform in 2011, followed by an Illumina-based method (version 1.1) in 2013^7,8^. A significant drawback of ChIP-exo 1.0 and 1.1 is their technical complexity compared to the lower resolution ChIP-seq assay. This has limited its broader adoption.

In an effort to simplify ChIP-exo library construction, version 2 (called ChIP-nexus) was developed in 2015^9^, in which the intermolecular 2nd adapter ligation was replaced by an intramolecular ligation. Despite this improvement, both version 1 and 2 of ChIP-exo remain technically difficult and costly. We therefore undertook a systematic effort to simplify the assay. This included a reduction in the number of enzymatic steps and alternative strategies for adapter ligation. The practical benefits of improved library construction include reduced costs, reduced processing time, and increased yield. We present multiple alternative versions because, in addition to each producing the expected resolution, each have particular trade-offs of advantages and limitations that may make one method more suitable for particular applications.

## Results

### ChIP-nexus (ChIP-exo 2.0) assessment

ChIP-nexus was published as an updated version of the original ChIP-exo protocol that reported increased efficiency of adapter ligation through use of CircLigase^9^. CircLigase catalyzes the self-circularization of single-stranded (ss) DNA and was used to reduce the number of intermolecular adapter ligation steps from two to one (Table 1). This reduction is achieved by putting both Illumina adapter sequences on a single oligonucleotide separated by a BamHI restriction site. Following library DNA circularization, the BamHI digestion creates linearized libraries that are suitable for DNA sequencing. We first examined the overall utility of ChIP-nexus (ChIP-exo 2.0) as a replacement for ChIP-exo 1.1.

**Table 1:** Comparison of steps used in each ChIP-exo assay version

**Fig. 1:**
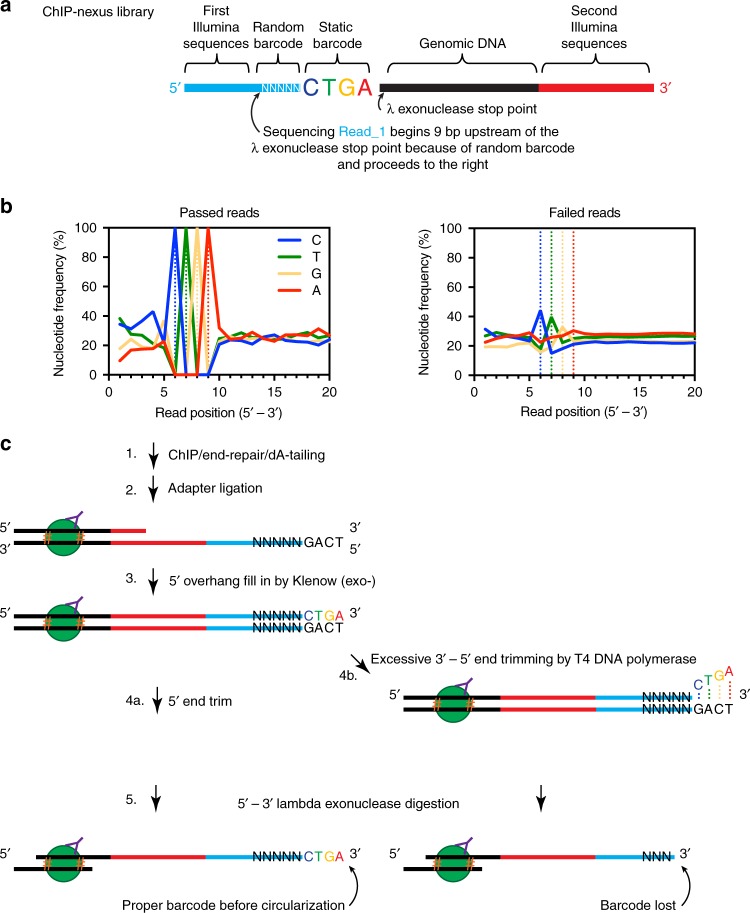
Evaluation of ChIP-nexus data. **a** Schematic of a completed ChIP-Nexus DNA library. **b** Nucleotide frequency at the 5′ end of the sequencing tags among tags that pass (left) or fail (right) the computational filter as defined previously^9^. **c** Proposed explanation for the pattern of nucleotide frequency observed in tags that fail to pass filter. Desired end-trimming produces blunt-end DNA as shown in steps 3 and 4a. Excessive trimming will produce a 5′ overhang in step 4b that would result in the pattern at the sequenced tag seen in (**b**). Orange hashtags represent protein-DNA cross-links

In a completed ChIP-nexus library, a five bp random barcode and a four bp static barcode are incorporated immediately 3′ to where sequencing begins and immediately 5′ to the lambda exonuclease stop point (Fig. 1a). These are the first nine nucleotides of the first sequencing read (Read_1, representing a ChIP-nexus “tag”), which are used to computationally remove PCR-duplicates and assess library quality. In evaluating the published ChIP-nexus data, we noticed that a significant number of sequencing tags (ranging from 20 to 95% for individual samples) were discarded because of poor barcode quality^9^. This represents a substantial loss of data. We next investigated the basis for this data loss. By definition, every sequencing read that passed the quality control filters contained the nucleotides CTGA in positions 6–9 of Read_1 (Fig. 1b, left panel). In reads that failed to pass filter, we observed a sequential loss of nucleotides in the static barcode (A>G>T>C decrease in peak amplitude, Fig. 1b, right panel). The start of this progression is internal to the completed library (i.e., not where sequencing begins), which is difficult to reconcile. However, these sequences reside at the end of the adapter prior to circularization. We therefore suggest two possible sources: (1) incomplete oligo synthesis (these sequences were synthesized last, making them the least efficiently incorporated), or (2) T4 DNA polymerase end-trimming that occurs immediately before lambda exonuclease treatment. End-trimming is intended to create blunt-end DNA via the strong 3' to 5' exonuclease activity of T4 DNA polymerase (Fig. 1c). Overdigestion would result in preferential loss of A>G>T>C as seen in Fig. 1b, and illustrated in step 4b of Fig. 1c. This analysis indicates that the ChIP-nexus assay results in a substantial loss of data. While deeper sequencing could compensate, this incurs a higher financial cost. Moreover, the amount of CircLigase used in comparison to traditional T4 DNA ligase has a ~10-fold increase in cost. ChIP-nexus also requires the additional enzymatic step of BamH1 digestion. We conclude that ChIP-nexus does not substantially improve the costs or technical difficulty of the ChIP-exo assay. In an effort to improve ChIP-exo, we revisited each step of library construction.

### Tn5 tagmentation-based ChIP-exo 3.x

To simplify the addition of adapters during library construction, we endeavored to replace DNA ligase with a hyperactive mutant Tn5 transposase^10^. This tagmentation reaction has been used to construct libraries for shotgun genome sequencing, chromatin accessibility (ATAC-seq), and ChIP-seq of transcription factors (ChIPmentation)^11–13^. In ChIPmentation, chromatin is first fragmented by sonication, then immunoprecipitated and tagmented while on the resin. Tn5 dimers bind a pair of 19 bp DNA recognition sequences. An optimized version of the recognition sequence has been incorporated into the Illumina Nextera sequencing adapters. The mutant Tn5 inserts one end of each 19 bp DNA recognition sequence into genomic DNA at reduced target sequence specificity. This fragments the chromatin.

In an effort to develop a cost-effective tagmentation-based ChIP-exo assay (version 3.x series), we constructed a Tn5 *E. coli* expression vector housing the E54K, E110K, P242A, and L372P mutations. These mutations create a hyperactive Tn5 that binds normally to its 19 bp recognition sequence, but has less sequence specificity for insertional targeting^14^. We also included an N-terminal His_6_-tag for purification purposes. The three-step purification produced a high-active enzyme that was >95% pure (Supplementary Fig. 1).

In the ChIP-exo 3.x series, immunoprecipitated chromatin was tagmented while on beads, as in ChIPmentation^12^. At this point, it was necessary to remove the spent Tn5 that remained bound to the product DNA^10^, while maintaining the protein-DNA cross-links and protein-antibody interaction (Supplementary Fig. 2a). This is distinct from tagmentation. We found that the reacted Tn5 could be stripped away using 500 mM guanidine hydrochloride buffer (Supplementary Fig. 2b). This allowed for efficient lambda exonuclease digestion equivalent to standard (version 1.1) ChIP-exo library construction^6^. Other chaotropic wash buffers were also successful (Supplementary Fig. 2c). ChIP-exo 3.x shortened the time for sample processing (Table 1), while maintaining library complexity (independent sampling events).

**Fig. 2:**
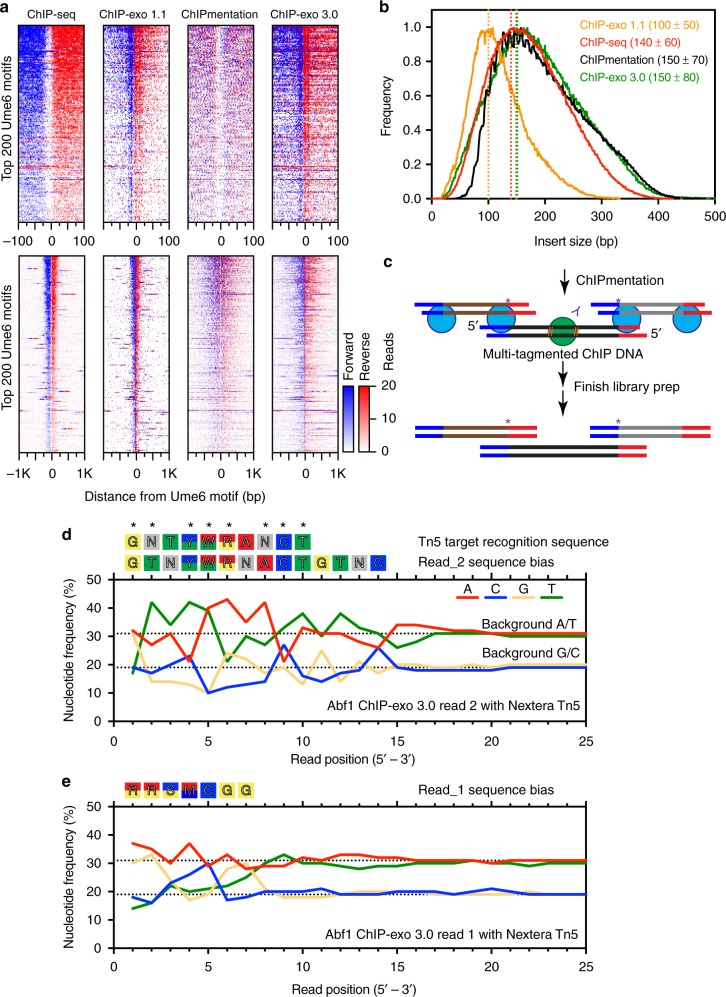
Tn5-based ChIP assays produce a high degree of sequence bias in reads. **a** Heatmaps comparing assay variants at the top 200 *S. cerevisiae* Ume6 motifs in a 200 bp (top) or 2 kb (bottom) window. Tag 5′ ends located on the motif strand are indicated in blue, and those located on the opposite strand are in red. Rows are linked and sorted (in all figures) based on motif-associated tag intensity derived from ChIP-exo 5.0 (our preferred method). **b** Frequency distribution of library insert sizes determined by paired-end sequencing for assay version shown in panel (**a**). Dotted lines indicates the modal insert size within each dataset. The numbers in parentheses represent the insert size mode plus/minus one standard deviation. **c** Proposed model of multi-tagmented DNA in ChIPmentation. Following tagmentation, Tn5 (blue spheres) does not dissociate, allowing the excess cut DNA (brown and gray lines) to remain noncovalently bound to the ChIP DNA (black lines with green sphere) through the end of library prep. This results in a mixture of forward and reverse strand tags at the original tagmentation sites that appear purple when viewed in a heatmap (purple asterisks). **d** Nucleotide frequency at the 5′ end of Read_2 sequencing tags (and thus not exonuclease digested) generated through tagmentation. Black dotted lines indicate the background nucleotide frequency of A/T (31% each) and G/C (19% each) content in *S. cerevisiae*. The observed sequence bias is displayed above the graph (IUPAC nomenclature). A sequence was deemed biased if the nucleotide frequency was more than 10% above background (A/T >34% or G/C >21%). The Tn5 insertional target recognition sequence^25^ is displayed above the observed sequence bias. Asterisks indicate positions that are consistent between the two sequences. **e** Nucleotide frequency at the 5′ end of Read_1 sequencing tags generated through exonuclease digestion. Exonuclease treatment masks the bias seen in panel (**d**), which is still present when considering tag yield (occupancy)

In the ChIP-exo 3.x series, second adapter ligation is potentially achieved via three alternative methods: A-tailing (3.0), a splint adapter (3.1), and a single-stranded adapter (3.2). These alternate methods of adapter ligation are developed below in the 4.x series. Based on that work, we produced tagmentation-based libraries using version 3.1. We tested ChIP-exo 3.1 on a set of yeast sequence-specific DNA binding proteins (Abf1, Reb1, and Ume6), all of which produced high quality data with ChIP-exo 1.x. We obtained the same qualitative results with all tested factors (Supplementary Fig. 3); but we will focus here on Ume6. Ume6 is a sequence-specific transcription factor that represses transcription of early meiotic genes through recruitment of chromatin remodelers^15^. Genome-wide binding of Ume6 was assayed by ChIP-seq, ChIP-exo 1.1, ChIPmentation, and ChIP-exo 3.1 (and also subsequent versions). The resulting tag 5′ ends were plotted around the top 200 bound Ume6 motifs (Fig. 2a, blue represents those on the reference motif strand, and red for those on the opposite strand). ChIP-exo 3.1 resulted in the same exonuclease stop sites at Ume6 motifs (vertical “stripes”) as seen with version 1.1 (Supplementary Fig. 4a). Thus, high resolution ChIP-exo can be conducted on tagmented chromatin.

### Limitations in ChIP-exo 3.1 and ChIPmentation

In ChIP-exo 3.1, we observed that a substantial proportion of tag 5′ ends mapped hundreds of bp beyond the core exonuclease stop sites, which was largely absent in version 1.1 (broad shouldering in Supplementary Fig. 4a). We surmised that this may reflect ineffective exonuclease digestion of a portion of the tagmented DNA molecules, whereas other molecules were digested effectively. Additional stringent washes of the chromatin did not improve the outcome. If the stringent washes failed to completely remove Tn5, then the residual Tn5 may be blocking exonuclease digestion. This makes ChIP-exo 3.1 relatively less efficient from a sequencing yield perspective, in that most specific tags were lower resolution than version 1.1.

Surprisingly, the broad shouldering in version 3.1 was at least as broad as in ChIP-seq and equivalent to ChIPmentation (red and blue flanks in Fig. 2a, lower set of panels; also Fig. 2b). Increased concentrations of Tn5/adapter complexes, within limits of practicality, did not appreciably reduce the broad shouldering (not shown). It was expected that the library size of ChIPmentation would be marginally shorter than ChIP-seq due to Tn5 fragmentation of the already-sonicated chromatin. We also expected ChIP-exo 3.1 to be ~50 bp shorter than ChIPmentation, due to shortening by the exonuclease. However, the main observed difference was an increase in abundance of larger fragments (Fig. 2b). We attribute this to preferential tagmentation of longer DNA molecules due to more opportunities for Tn5 binding^16^. Many short fragments may have gone unreacted. Consequently, tagmentation created a bias towards larger library fragment size, which may create a bias in binding site detection.

A second surprising finding was that, unlike version 1.1 and ChIP-seq, Tn5-based (ChIPmentation) assays produced substantial amounts of tag 5′ ends that mapped downstream (more 3′) of the reference motif (Supplementary Fig. 4a, b). Thus, the ChIPmentation heatmap did not produce a segregated “blue/red” left/right pattern of tag 5′ ends, as seen in the ChIP-seq and ChIP-exo 1.1 heatmaps (Fig. 2a). We suggest that this may be due to multiple Tn5 hits occurring on the same ChIP DNA molecule, but with the multiple DNA fragments being held together noncovalently via Tn5-DNA product complexes (Fig. 2c). This would allow tag 5′ ends to map downstream of the cross-link. In contrast, in normal ChIP-seq the 5′ ends of the double-stranded DNA reside on opposite sides of the ChIP peak (left panel in Fig. 2a). Thus, when analyzed by tag 5′ ends, ChIPmentation produced a resolution below that of ChIP-seq.

To test whether the tagmentation issues identified above were particular to our Tn5 preparation or are also present in commercially-available Tn5, we used the Nextera DNA Library Preparation Kit. For these particular experiments, we compared the two preparations against the sequence-specific DNA binding protein Abf1. Significant shoulders were somewhat reduced, but still prominent with the commercial Tn5 (Supplementary Fig. 4c).

Although the hyperactive Tn5 was engineered to be less sequence-specific in insertional target site selection, we found that both our in-house Tn5 preparation and the Nextera Tn5 retained a strong cleavage preference for the wildtype target recognition sequence (Fig. 2d). This targeting bias significantly skewed the nucleotide frequency at the 5′ end of tagmented sequencing reads, as seen previously^17^. The lambda exonuclease treatment eliminated most of the bias in ChIP-exo 3.1 reads (Fig. 2e), but this only masked the intrinsic Tn5 cleavage bias, which skews the quantitative occupancy level of ChIP locations. Taken together, while ChIP-exo 3.1 is technically simpler than 1.x/2.0, and provides equivalent location resolution, it has some undesirable qualities in library bias and resolution.

### ChIP-exo 4.x development (single-stranded DNA ligation)

To further reduce the number of steps and increase efficiency for standard adapter ligation, we turned to strategies employing ssDNA ligation. In ChIP-exo 1.x, double-stranded ChIP DNA is blunt-ended, then A-tailed prior to ligation of the first adapter by T4 DNA ligase. This reaction is inefficient, likely due to the numerous (four) enzymatic steps. However, efficient intermoleculer ligation has been described between the 3′ ends of ssDNA and the 5′ ends of a single-stranded adapter oligo using either CircLigase^18^ or T4 DNA ligase^19^.

To incorporate ssDNA ligation into ChIP-exo, while retaining use of the more cost-effective T4 DNA ligase (compared to CircLigase), we rearranged the order of enzymatic steps such that lambda exonuclease digestion occurred before DNA ligation (Table 1). We then exploited the polarity of the resected 5′ ends generated by lambda exonuclease to direct end-specific hybridization-based DNA ligation (Supplementary Fig. 5a). In this case, the Read_1 adapter was a ssDNA oligo that contained a random pentamer at its 3′ end. Hybridization of the random pentamer to the ssDNA that was complementary to the resected strand, and adjacent to the exonuclease stop site produced efficient ligation. In this scheme, the random pentamer sequence represents the first five positions of the sequencing read, making the exonuclease stop site appear 5 bp more 5′ than in other versions of ChIP-exo. This is version 4.0. The ligation scheme differs from other ssDNA ligation descriptions in being conducted on immobilized DNA rather than in solution, and involving adapter ligation to the 5′ end of genomic DNA rather than its 3′ end.

In an alternative scenario (ChIP-exo 4.1), we conducted the first ligation to the 3′ ssDNA end of the resected ChIP-exo DNA, while the DNA remained immobilized on resin (Supplementary Fig. 5b). This was performed by using a double-stranded Read_2 adapter having a random 3′ ssDNA pentamer overhang. Upon hybridization of this “splint” to the terminal five nucleotides of ChIP-exo ssDNA 3′ end, T4 DNA ligation of the complementary adapter strand to the ChIP-exo ssDNA 3′ end proceeded efficiently.

In the ChIP-exo 4.x series, following reversal of the formaldehyde cross-linking, the second adapter is attached using splint ligation of proper polarity (Read_2 adapter for 4.0, and Read_1 adapter for 4.1). By using random pentamers to guide adapter ligation instead of standard A-tailing, ChIP-exo 4.x eliminates nine enzymatic steps and nearly six hours of hands-on time from standard ChIP-exo. The two ligation steps could, in principle, be conducted simultaneously with the maintenance of polarity. However, in practice we have found an unacceptably high level of adapter dimer contamination occurred if a clean-up step was not incorporated between adapter ligation steps. Thus, we did not adopt this option, and instead separated the ligation steps.

**Fig. 3:**
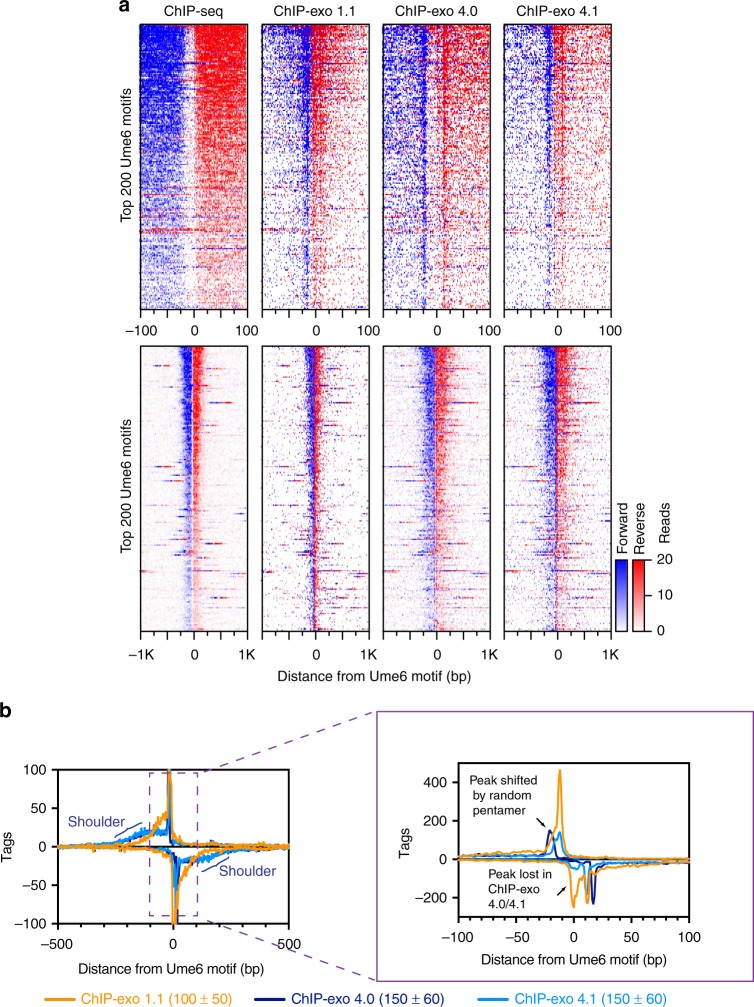
ChIP-exo 4.0/4.1 display increased shouldering at the binding site compared to version 1.1. **a** Heatmaps comparing assay versions at the top 200 Ume6 motifs in a 200 bp (top) or 2 kb (bottom) window. **b** Composite plots of assay versions in a 1 kb window (left) and zoomed to 200 bp (right). The 1 kb window highlights the increased shouldering observed in ChIP-exo 4.0/4.1. The zoomed view highlights that peaks in ChIP-exo 4.0 are shifted 5 bp away from the motif center due to incorporation of the random pentamer; and the peak observed in ChIP-exo 1.1 at the motif midpoint was absent from the ChIP-exo 4.0/4.1 pattern

Much like ChIP-exo 3.1, we found that 4.x provided high resolution patterning of factor binding, but also contained significant amounts of low-resolution shouldering, presumably from incomplete exonuclease digestion (Fig. 3a and Supplementary Fig. 3). Given the caveat that ChIP-exo peaks detected in version 4.0 are shifted five bp further away from the motif midpoint, the ChIP-exo 4.x composite plots produced the same outer peaks (exonuclease stops) as ChIP-exo 1.1/3.1 (Fig. 3a, blue/red vertical stripes are farther apart in ChIP-exo 4.0 panel). However, a secondary inner peak on the motif-complementary strand in the Ume6 pattern was greatly reduced in version 4.x, but not 1.1/3.1 (Fig. 3b and Supplementary Fig. 4a). We have no definitive explanation for this difference, although it might relate to an altered ligation efficiency that occurs at or near a particular motif sequence or cross-link. Nevertheless, ChIP-exo 4.x represent two highly streamlined versions of ChIP-exo, with the limiting caveat of potentially not detecting all peaks within a family of peaks that define an individual binding location. This limitation did not exist with the other tested factors (Supplementary Fig. 3).

### ChIP-exo 5.0 development

While ssDNA ligation in versions 4.x significantly improved technical aspects of ChIP-exo, the relative level of undesirable “shouldering” (undigested ChIP DNA) was greater when compared to ChIP-exo 1.x. The low shouldering in ChIP-exo 1.x might be due to it having the first ligation step performed prior to exonuclease digestion, as this is the major distinction between versions 1 and 4. Conceivably, the early enzymatic steps may have created or selected for DNA molecules that are competent for exonuclease digestion. We therefore returned to the processing order specified in ChIP-exo 1.1, but tested the assumption that every step in ChIP-exo 1.x is required, since those steps were based on theoretical expectations, rather than actual experimental testing.

The first enzymatic step in ChIP-exo is to create blunt ends from DNA fragmented by sonication, using T4 DNA polymerase (Table 1). Since T4 DNA polymerase possesses both 5′ to 3′ synthesis and 3′ to 5′ exonuclease activities, the reaction was carried out at 12 °C to balance these opposing activities. However, from our analysis of ChIP-exo 2.0 (Fig. 1b, c), we were concerned that even at the lower temperature T4 DNA polymerase would not produce more ligatable blunt ends through synthesis than it eliminated through exonuclease activity. Consequently, in ChIP-exo 5.0 all polishing steps were removed. A-tailing by Klenow was maintained as it restored “shouldering” to acceptable levels, as seen in 1.x.

**Fig. 4:**
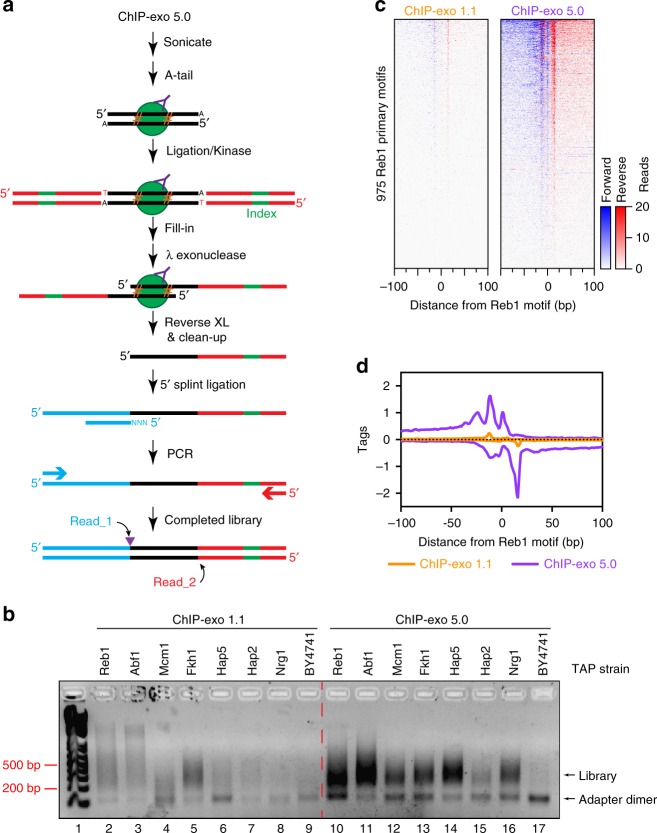
ChIP-exo 5.0 increases library yield. **a** Schematic of ChIP-exo 5.0. The purple triangle indicates the location of the Read_1 start site, which is also the λ exonuclease stop site. **b** 2% agarose gel of the electrophoresed library following 18 cycles of PCR for various *S. cerevisiae* transcription factors assayed by ChIP-exo 1.1 or 5.0. Following ChIP, the sample was split and libraries prepared using the indicated protocols. After splitting the sample, each reaction contained a 50 ml cell equivalent (OD_600_ = 0.8) of yeast chromatin, which is five-fold less than the amount optimized for ChIP-exo 1.1. ChIP-exo 5.0 produced greater library yield for all samples. **c** Heatmaps comparing ChIP-exo 1.1 and 5.0 at the 975 Reb1 primary motifs in a 200 bp window. **d** Composite plot of data from panel (**c**)

The next two steps after A-tailing involved T4 Kinase and T4 DNA ligase. Since both work well in the same buffer, we combined them into a single step, allowing both the ChIP DNA and adapter 5′ ends to be phosphorylated and ligated (despite the oligos being synthesized with a 5′ phosphate). The ligation buffer was altered to include polyethylene glycol, which as demonstrated elsewhere^9^, increased yield and decreased incubation times. We also removed RecJ_f_ exonuclease digestion. Its original purpose was to eliminate nonspecific ssDNA contaminants that might arise from lambda exonuclease digestion of contaminating double-stranded DNA. However, this step had no discernible impact on library quality. As a result of these improvements, five enzymatic steps and four hours of incubation time were eliminated from this part of the ChIP-exo 1.1 protocol. The remaining steps were performed as in ChIP-exo 4.1 (Table 1). The entirety of this streamlined procedure is ChIP-exo 5.0 (Fig. 4a).

With ChIP-exo 5.0, the same high quality libraries and data as 1.1 were obtained with only five enzymatic steps compared to the original thirteen. The reduction in steps also greatly increased library yield when the same ChIP reactions were split and libraries were constructed using either the 1.1 or 5.0 versions of the protocol (Fig. 4b). The increased yield of ChIP-exo 5.0 also led to a higher rate of successful ChIPs for low-abundance factors such as Mcm1, Fkh1, Hap5, Hap2, and Nrg1. When equal amounts of library were sequenced, version 5.0 produced 10 times more mapped tags than version 1.1, after removal of PCR duplicates (Fig. 4c). ChIP-exo 5.0 produced robust Reb1 data, even though the chromatin input in the immunoprecipitation was reduced five-fold relative to the published ChIP-exo 1.1 protocol. With the same amount of chromatin, ChIP-exo 1.1 barely registered Reb1 binding (Fig. 4d).

### Application to low amounts of mammalian cells

**Fig. 5:**
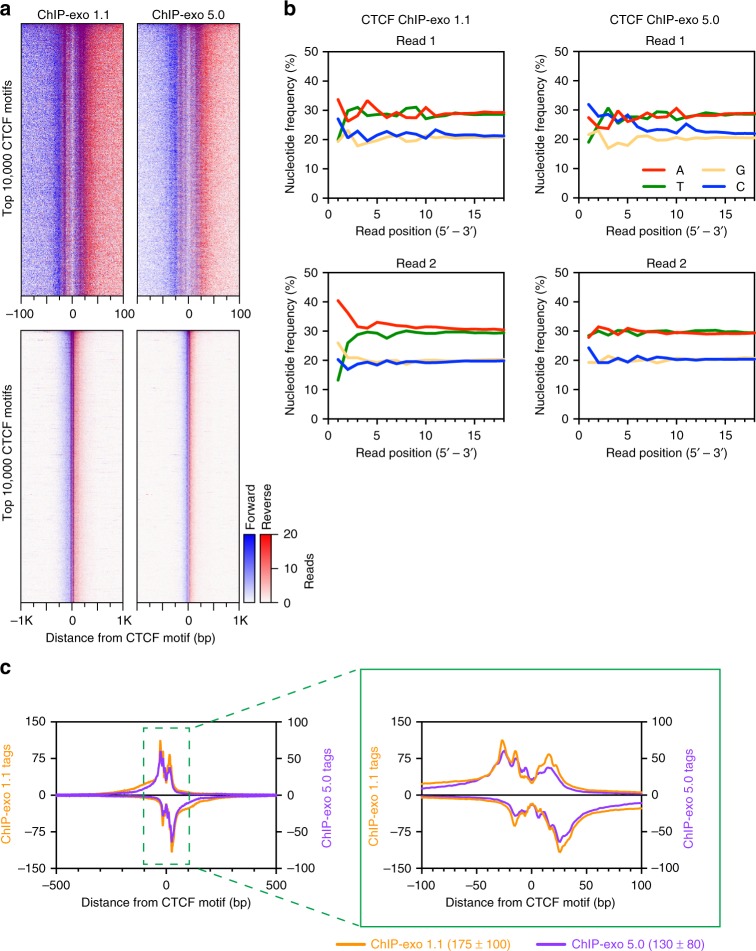
ChIP-exo 5.0 produced the same quality data as ChIP-exo 1.1. **a** Heatmaps comparing ChIP-exo 1.1 and ChIP-exo 5.0 at the top 10,000 *H. sapiens* CTCF motifs in a 200 bp (top) or 2 kb (bottom) window. **b** Comparison of the nucleotide frequency at the 5′ end of the sequencing tags for ChIP-exo 1.1 and ChIP-exo 5.0 in CTCF datasets. Read_1 5′ end is the product of exonuclease digestion. Read_2 5′ end is the product of ligation following A-tailing of sonicated ends. **c** Composite plots of data in panel (**a**) in a 1 kb window (left) and zoomed to 200 bp (right)

To demonstrate that the advantages of ChIP-exo 5.0 are not exclusive to yeast samples, we performed ChIP-exo 5.0 for CTCF with human K562 cells. Compared to ChIP-exo 1.1, ChIP-exo 5.0 produced equivalently high resolution CTCF exonuclease stop sites (Fig. 5a), with relatively little nucleotide bias near the ligated ends (Fig. 5b), and nearly identical composite plots (Fig. 5c). In every cell type tested, we have found that ChIP-exo 5.0 is a strict improvement over the ChIP-exo 1.1 method.

**Fig. 6:**
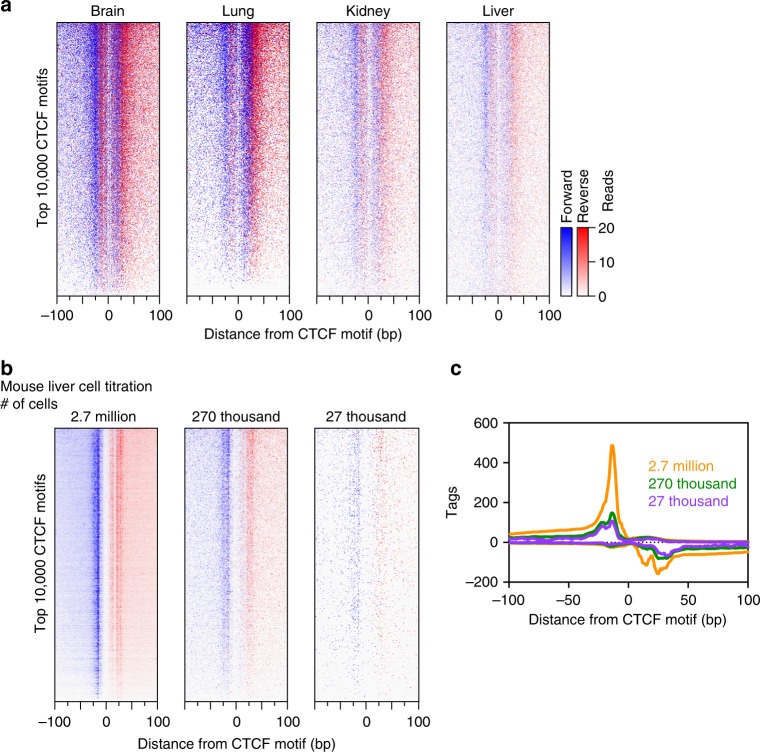
ChIP-exo 5.0 data from mouse organs. Heatmaps of ChIP-exo 5.0 CTCF (**a**) across multiple organs or (**b**) in a liver cell titration at the top 10,000 *M. musculus* CTCF motifs in a 200 bp window. Rows were sorted by the average rank order of the four datasets in (**a**) by tag counts in a 200 bp window centered on the motif midpoint. Heatmaps are linked. **c** Composite plot of data in (**b**)

Using ChIP-exo 5.0, we efficiently detected CTCF binding across a variety of mouse organ tissues (brain, lung, kidney, and liver, Fig. 6a). Site identification, signal strength, and exonuclease patterning were equivalent to that seen in tissue culture. This demonstrates the utility of ChIP-exo 5.0 in defining site-specific DNA binding at high resolution in animal organs. Furthermore, we are able to detect site-specific CTCF binding with as few as 27,000 cells (Fig. 6b, c), albeit at lower library complexity. Thus, in principle ChIP-exo is applicable to small amounts of living specimens, as low as 200 µg wet weight from tissue biopsies. However, optimal yields are likely to require at least 2 mg of tissue (~250,000 cells).

## Discussion

ChIP-seq was developed as a powerful method for determining nucleosome-bound and transcription factor-bound regions of the genome^4,5^. ChIP-exo 1.0 built upon that utility by taking advantage of factor-specific cross-linking patterns within each DNA binding event to achieve the following: (1) improve signal-to-noise detection, thereby providing more a comprehensive set of bound locations, (2) elucidate the positional organization of proteins within a complex, and (3) detect alternative binding modes. Technical difficulty and sequencing platform restriction of the original assay may have limited broader adoption. Version 1.1 brought ChIP-exo to the Illumina platform. Version 2.0 (ChIP-nexus) provided some simplification by eliminating two enzymatic steps, but required multiple ligations steps and an extra restriction endonuclease step. Version 2.0 also resulted in some data loss, possibly due to unintended enzymatic loss of barcode information.

ChIP-exo 3.0, presented here, takes advantage of one-step adapter attachment afforded by Tn5 tagmentation. This version is technically simpler than all prior versions of ChIP-exo and retains ultra-high resolution. However, we found it to have diminished performance characteristics, including increased sequence-specificity of targeting that produces partially biased libraries. It produces “shouldering”, which is essentially a signal distribution pattern that is equivalent to ChIP-seq and ChIPmentation. Thus, version 3.0 may be useful where assay simplicity is paramount and a blend of ChIP-exo and ChIP-seq signal patterning is acceptable.

ChIP-exo 4.x was developed to streamline library construction in a way that avoided the biases of Tn5 (version 3.x). 4.x involves ligating the first adapter after lambda exonuclease digestion, with version 4.0 ligating a ssDNA adapter (corresponding to Read_1) to the resected ChIP-exo DNA. Version 4.1 uses a splint in ligating to the non-resected end (Corresponding to Read_2). Both use a splint in the second ligation step. These versions are both technically quite simple and lack the bias of Tn5. However, both displayed shouldering as seen in version 3.0. Thus, 4.x versions are technically the simplest of all versions, and may be the method of choice where ChIP-exo patterning is desired, but where some level of lower-resolution ChIP-seq quality signal can be tolerated. A caveat of ChIP-exo 4.0 is that since a ligation event takes place within a few bp of a protein-DNA cross-link, it may in some cases create steric exclusion, making certain cross-linking points within a binding event go undetected.

ChIP-exo 5.0 was developed to alleviate shouldering (ChIP-seq quality signal) arising in versions 3.x and 4.x. In total, thirteen enzymatic steps were reduced to five. ChIP-exo 5.0 is the most suitable assay to maximize signal concentration from ChIP-exo patterning. While we have not identified which aspects of versions 3.x/4.x produce shouldering, it is our assessment that the initial A-tailing and adapter ligation in versions 1 and 5 may select for ChIP DNA molecules that are subsequently digestible by lambda exonuclease, thereby eliminating shoulders. ChIP-exo 3.0, 4.0, and 4.1 did not incorporate the additional streamlined steps used in version 5.0, although such steps may be applied to these versions, such as in 3.1.

The value of ChIP-exo over ChIP-seq is the insight provided by exonuclease patterning, and the increased signal:noise that adds greater confidence to location calling. Therefore, we favor the streamlined ChIP-exo 5.0. All versions of ChIP-exo in principle produce essentially the same lambda exonuclease pattern, so switching across any version of the assay, as might occur in an extended series of experiments, should have little impact on the qualitative conclusions drawn. Since all versions of ChIP-exo are a derivative of ChIP-seq, ChIP-exo data can be converted to ChIP-seq data. In fact, Read_2 from our paired-end sequencing of ChIP-exo libraries is essentially a ChIP-seq signal. Therefore, even in our most highly refined ChIP-exo protocol, a pure ChIP-seq signal is separately produced and available for analysis. We use it (Read_2) to improve tag mappability, and identify PCR duplicates (as it has a heterogeneous 5′ end). Our ChIP-exo assays are performed in 8-well strip tubes using multichannel pipettors. This allows for significant high-throughput scale-up. These refined ChIP-exo assays, particularly version 5.0, may be highly suitable for large genome-wide mapping projects^20^. The efficiency and simplicity of the new ChIP-exo assay makes detection of genomic interactions in mammalian organs and tissue feasible, including tissue biopsies that may have relatively few cells.

## Methods

### Antibodies

Rabbit IgG (Sigma) conjugated to Dynabeads was used against TAP-tagged strains in which the TAP-tag containing Protein A was the target. Millipore 07–729 antibody was used against K562 samples targeting CTCF.

### Tn5 purification

A custom construct of Tn5 E54K E110K P242A L372P^14^ in a pET-45b( + ) vector was ordered (GenScript) to express hyperactive Tn5 with an N-terminal His_6_-tag. The plasmid is available at Addgene (Addgene ID: 112112). BL21(DE3) competent *E. coli* cells (New England Biolabs) were transformed and a single colony was grown at 37 °C to an OD_600_ of 0.4 in 500 ml of LB + 50 µg/ml ampicillin + 30 µg/ml chloramphenicol. Cells were transferred to a 25 °C incubator and induced with 0.5 mM isopropyl-β-D-galactopyranoside for 4 h. The cells were collected by centrifugation, washed once with ST Buffer, and the cell pellet was flash frozen in liquid nitrogen.

Tn5 was purified as previously described^10^, with few modifications. Briefly, cells were resuspended in 10 volumes (ml/g) of TEGX100 Buffer (20 mM Tris-HCl, pH 7.5, 100 mM NaCl, 1 mM EDTA, 10% glycerol, 0.1% Triton-X 100) containing CPI and 100 µM phenylmethylsulfonyl fluoride and lysed by incubation with lysozyme (Sigma; 1 mg / 1 g of cell pellet) at room temperature for 30 min. The lysate was centrifuged at 20,000 × g for 20 min at 4 °C, and the supernatant was precipitated with 0.25% polyethyleneimine (Sigma) and centrifuged at 10,000 × g for 15 min. The supernatant was then precipitated with 47% saturation ammonium sulfate (0.28 g/ml) over a 30 min incubation, and then centrifuged at 20,000 × g for 15 min.

The pellet was then resuspended in 50 ml of Nickel Affinity Load Buffer (50 mM potassium phosphate, pH 7.4, 50 mM KCl, 20% glycerol) and loaded on a HisTrap HP column (GE Healthcare; 5 ml) at 1.5 ml/min equilibrated with the same buffer. The column was sequentially washed with Wash Buffer I (50 mM potassium phosphate, pH 7.4, 1 M KCl, 50 mM imidazole, 20% glycerol), Wash Buffer II (50 mM potassium phosphate, pH 7.4, 500 mM KCl, 50 mM imidazole, 20% glycerol), and then Tn5 was eluted with Nickel Affinity Elution Buffer (50 mM potassium phosphate, pH 7.4, 500 mM KCl, 500 mM imidazole, 20% glycerol) at 2 ml/min. The eluate was diluted to 300 mM KCl with Dilution Buffer (50 mM potassium phosphate, pH 7.4, 20% glycerol), and the final volume adjusted to 50 ml with TEGX300 Buffer (20 mM Tris-HCl, pH 7.5, 300 mM NaCl, 1 mM EDTA, 10% glycerol, 0.1% Triton-X 100).

Next, the sample was loaded on a HiTrap Heparin HP column (GE Healthcare; 1 ml) equilibrated with TEGX300 at 1 ml/min. After washing with 5 column volumes of buffer, a 10-ml linear (300 mM to 1.2 M) NaCl gradient was run to elute. Tn5 eluted from the column at approximately 600 mM NaCl. Fractions containing the main elution peak were combined (3.5 ml) and dialyzed overnight against TEGX300 Buffer containing 30% glycerol, and then stored at −80 °C.

### Yeast chromatin preparation

TAP-tagged *Saccharomyces cerevisiae* strains (Open Biosystems) were grown in 500 ml of yeast peptone dextrose media to an OD_600_ = 0.8 at 25 °C. Cells were cross-linked with formaldehyde at a final concentration of 1% for 15 min at room temperature, and quenched with a final concentration of 125 mM glycine for 5 min. Cells were collected by centrifugation, and washed in 1 ml of ST Buffer (10 mM Tris-HCl, pH 7.5, 100 mM NaCl) at 4 °C and split into two aliquots. The cells were pelleted again, the supernatant was removed, and the pellet was flash frozen.

A 250 ml culture aliquot was lysed in 750 µl of FA Lysis Buffer (50 mM Hepes-KOH, pH 7.5, 150 mM NaCl, 2 mM EDTA, 1% Triton, 0.1% sodium deoxycholate, and CPI) and 1 ml volume of 0.5 mm zirconia/silica beads by bead beating in a Mini-Beadbeater-96 machine (Biospec) for three cycles of 3 min on/5 min off cycles (Samples were kept on ice during the off cycle). The lysate was transferred to a new tube and microcentrifuged at maximum speed for 3 min at 4 °C to pellet the chromatin. The supernatant was discarded, and the pellet was resuspended in 750 µl of FA Lysis Buffer supplemented with 0.1% SDS and transferred to a 15 ml polystyrene conical tube. The sample was then sonicated in a Bioruptor (Diagenode) for 15 cycles with 30 s on/off intervals to obtain DNA fragments 100 to 500 bp in size. One ChIP-exo assay processed the equivalent of 50 ml cell culture (~6 × 10^8^ cells). This represents a convenient amount rather than a minimum amount needed.

### K562 chromatin preparation

Human chronic myelogenous leukemia cells (K562, ATCC) were maintained between 1 × 10^5^ and 1 × 10^6^ cell/ml in DMEM media supplemented with 10% fetal bovine serum at 37 °C with 5% CO_2_. Cells were washed with PBS (8 mM Na_2_HPO_4_, 2 mM KH_2_PO_4_, 150 mM NaCl, and 2.7 mM KCl), then cross-linked with formaldehyde at a final concentration of 1% for 10 min at room temperature, and quenched with a final concentration of 125 mM glycine for 5 min. The supernatant was removed, and the cells were resuspended in 1 ml PBS to wash. Cells were aliquoted to contain 100 million cells, centrifuged, the supernatant was removed, and the pellet was flash frozen.

A 100 million cell aliquot (for use in multiple ChIPs) was lysed in 500 µl (10 mM Tris-HCl, pH 8.0, 10 mM NaCl, 0.5% NP40, and complete protease inhibitor (CPI, Roche)) by incubating on ice for 10 min. The lysate was microcentrifuged at 2500 rpm for 5 min at 4 °C. The supernatant was removed, the pellet resuspended in 1 ml (50 mM Tris-pH 8.0, 10 mM EDTA, 0.32% SDS, and CPI), and incubated on ice for 10 min to lyse the nuclei. The sample was diluted with 600 µl of immunoprecipitation dilution buffer (IP Dilution Buffer: 20 mM Tris-HCl, pH 8.0, 2 mM EDTA, 150 mM NaCl, 1% Triton X-100, and CPI) to a final concentration of (40 mM Tris-HCl, pH 8.0, 7 mM EDTA, 56 mM NaCl, 0.4% Triton-X 100, 0.2% SDS, and CPI), and sonicated with a Bioruptor (Diagenode) for 10 cycles with 30 s on/off intervals to obtain DNA fragments 100 to 500 bp in size.

### Mouse tissue preparation

16 week old adult male mouse brain, lung, liver, and kidney tissues were generously provided by Dr. Yanming Wang Mouse tissues were processed at 100 mg and chromatin was generated as previously described^21^ with minor modifications. In brief, 100 mg of mouse tissue was minced into pieces on ice, fixed with 1% formaldehyde for 10 min, and then quenched with a final concentration of 125 mM glycine for 5 min. Cells were spun, washed with PBS, and then resuspended in 1 mL of cold Farnham cell lysis buffer (20 mM Tris-HCl, pH 8.0, 85 mM KCl, 0.5% NP-40, 0.5% Triton X-100, and CPI). Cells were then incubated on a rototorque for 20 min at 4 C. Isolated nuclei were isolated by spun down and resuspended in RIPA nuclear lysis buffer (1 × PBS, 1% NP-40, 0.5% NaDeoxycholate, 0.5% SDS, and CPI). Nuclei were then sonicated with a Bioruptor (Diagenode) for 10 cycles with 30 s on/off intervals to generate DNA in the 100–500 bp size range. Liver cell numbers were estimated as previously described^22^, using a conversion factor of 1 mg tissue wet weight equals ~250,000 cells.

### Chromatin immunoprecipitation

A 50 ml culture-equivalent of yeast or 10 million cell-equivalent of K562 chromatin was diluted to 200 µl with IP Dilution Buffer and incubated overnight at 4 °C with the appropriate antibody. A 10 µl bed volume of IgG-Dynabeads was added to the yeast samples; and 3 µg of anti-CTCF antibody with a 10 µl slurry-equivalent of Protein A Mag Sepharose (GE Healthcare) was added to the K562 samples.

### ChIP-exo 1.1

ChIP-exo 1.1 was performed as previously described^7,8,23^. In brief, the following enzymatic steps were carried out with immunoprecipitated chromatin still on the resin with multiple salt washes between each step: T4 DNA polymerase end polishing, T4 polynucleotide kinase, Klenow fragment A-tailing, T4 DNA ligase-mediated Read_2 adapter ligation, phi29 DNA polymerase fill-in, second T4 polynucleotide kinase, lambda exonuclease digestion, and RecJ_f_ exonuclease digestion. Following overnight reverse cross-linking and Proteinase K treatment, the following steps were carried out in solution: phi29 primer extension, second Klenow fragment A-tailing, T4 DNA ligase-mediated Read_1 adapter ligation, and PCR.

### ChIP-exo 3.0 and 3.1 (tagmentation-based version)

After immunoprecipitation, the following steps were carried out on the resin. To assemble the Transposase, Tn5 was incubated in a 10 × Transposase Mix containing the following components and incubated for 30 min at room temperature: 12.5 µM Tn5, 50% glycerol, and 7.5 µM adapter (NexA2/ME comp). See Supplementary Table 1 for oligonucleotide sequences used in this study.

The ChIP material on resin was washed sequentially with FA Lysis Buffer, NaCl Buffer (50 mM HEPES-KOH, pH 7.5, 500 mM NaCl, 2 mM EDTA, 1% Triton-X 100, 0.1% sodium deoxycholate), LiCl Buffer (100 mM Tris-HCl, pH 8.0, 500 mM LiCl, 1% NP-40, 1% sodium deoxycholate), and 10 mM Tris-HCl, pH 8.0 at 4 °C.

The tagmentation reaction (30 µl) containing: 20 mM Tris-HCl, pH 7.5, 5 mM MgCl2, 10% dimethylformamide, and 1 × Tagmentation Mix from Step 1 (final concentration: 1.25 µM Tn5, 5% glycerol, 750 nM adapter) was incubated for 30 min at 37 °C. Following incubation, the resin was washed twice with Guanidine-hydrochloride Buffer (50 mM Tris-HCl, pH 7.5, 500 mM guanidine-hydrochloride, 2 mM EDTA, 1% Triton-X 100, 0.1% sodium deoxycholate) for 5 min at 37 °C, then once with LiCl Buffer and 10 mM Tris-HCl, pH 8.0 at 4 °C.

The fill-in reaction (30 µl) containing: 10 U phi29 polymerase (NEB), 1 × phi29 reaction buffer (NEB), 2 × (200 µg/ml) BSA, and 165 µM dNTPs was incubated for 20 min at 30 °C; then washed with 10 mM Tris-HCl, pH 8.0 at 4 °C. The kinase reaction (30 µl) containing: 10 U T4 PNK (NEB), 1 × T4 DNA Ligase Buffer (NEB), and 2 × BSA was incubated for 15 min at 37 °C; then washed with 10 mM Tris-HCl, pH 8.0 at 4 °C. The λ exonuclease digestion (100 µl) containing: 20 U λ exonuclease (NEB), 1 × λ exonuclease reaction buffer (NEB), 0.1% Triton-X 100, and 5% DMSO was incubated for 30 min at 37 °C; then washed with 10 mM Tris-HCl, pH 8.0 at 4 °C. The RecJ_f_ exonuclease digestion (100 µl) containing: 75 U RecJ_f_ exonuclease (NEB), 2 × NEBuffer 2, 0.1% Triton-X 100, and 5% DMSO was incubated for 30 min at 37 °C; then washed with 10 mM Tris-HCl, pH 8.0 at 4 °C.

DNA was eluted from the resin, and reverse cross-linking and Proteinase K treatment were performed (40 µl) containing: 30 µg Proteinase K, 25 mM Tris-HCl, pH 7.5, 2 mM EDTA, 200 mM NaCl, and 0.5% SDS incubated for 16 h at 65 °C. The supernatant was then transferred to a new tube and purified with Agencourt AMPure magnetic beads (Beckman Coulter) following manufacturer's instructions and using 1.8 × volume of AMPure slurry added to the DNA volume (72 µl).The sample was eluted from the AMPure beads in 10 µl of water, and the following enzymatic steps were carried out in solution.

Adapter ligation (version 3.0): for the primer extension reaction (total reaction volume 20 µl); to the resuspended sample was added 1 × phi29 reaction buffer, 2 × BSA, 100 µM dNTPs, and 0.5 µM ME sequence oligonucleotide (total 9 µl) and incubated for 5 min at 95 °C, then 10 min at 45 °C to allow the oligo time to anneal. The sample was shifted to 30 °C before adding 10 U phi29 polymerase (1 µl) and incubating for 20 min at 30 °C; then for 10 min at 65 °C to inactivate, and shifted to 37 °C.

For the A-tailing reaction (total reaction volume 30 µl); to the primer extension reaction was added 10 U Klenow Fragment, -exo (NEB), 1 × NEBuffer 2, 100 µM dATP (total 10 µl) and incubated for 30 min at 37 °C, then for 20 min at 75 °C to inactivate, and shifted to 25 °C. For the second adapter ligation reaction (total reaction volume 40 µl); to the A-tailing reaction was added 2,000 U T4 DNA ligase (enzymatics), 1 × NEBNext Quick Ligation Buffer (NEB), 375 nM adapter (ExA1-58/13) and incubated for 1 h at 25 °C.

Splint ligation (version 3.1): for adapter ligation (40 µl); to the resuspended DNA was added 1,200 U T4 DNA ligase, 1 × T4 DNA Ligase Buffer, 375 nM adapter (ExA1-58/ExA1-SSL_N5) and incubated for 1 h at 25 °C.

Both version 3.0 and 3.1: the ligation reaction was then purified with AMPure beads and resuspended in 15 µl of water. The sample was then amplified via PCR. For PCR amplification (total reaction volume 40 µl); to the resuspended DNA was added 2 U Phusion Hot Start polymerase (Thermo scientific), 1 × Phusion HF Buffer (Thermo scientific), 200 µM dNTPs, 500 nM each primer (P1.3 and NexA2-iNN) and amplified for 18 cycles (20 s at 98 °C denature, 1 min at 52 °C annealing, and 1 min at 72 °C extension). A quarter of the reaction was amplified for an additional six cycles (24 total) and the presence of libraries was determined by electrophoresis on a 2% agarose gel.

Size selection: 200–500 bp PCR products were gel-purified from a 2% agarose gel using the QIAquick Gel Extraction Kit (Qiagen).

### ChIP-exo 4.0 and 4.1 (single-strand DNA ligation versions)

After immunoprecipitation, the following steps were carried out on the resin. The ChIP material on resin was washed sequentially with FA Lysis Buffer, NaCl Buffer, LiCl Buffer, and 10 mM Tris-HCl, pH 8.0 at 4 °C. The end repair reaction (50 µl) containing: 7.5 U T4 DNA polymerase (NEB), 2.5 U DNA Polymerase I (NEB), 25 U T4 PNK, 1 × T4 DNA Ligase Buffer, and 390 µM dNTPs was incubated for 30 min at 12 °C; then washed with 10 mM Tris-HCl, pH 8.0 at 4 °C. The λ exonuclease digestion (100 µl)containing: 20 U λ exonuclease, 1 × λ exonuclease reaction buffer, 0.1% Triton-X 100, and 5% DMSO was incubated for 30 min at 37 °C; then washed with 10 mM Tris-HCl, pH 8.0 at 4 °C. The RecJ_f_ exonuclease digestion (100 µl)containing: 75 U RecJ_f_ exonuclease, 2 × NEBuffer 2, 0.1% Triton-X 100, and 5% DMSO swas incubated for 30 min at 37 °C; then washed with 10 mM Tris-HCl, pH 8.0 at 4 °C.

First adapter ligation: ssDNA ligation (version 4.0, 40 µl): for adapterion ligation 1200 U T4 DNA ligase, 1 × T4 DNA Ligase Buffer, and 375 nM single-strand adapter (ExA1-58-N5) was incubated for 1 h at 25 °C; then washed with 10 mM Tris-HCl, pH 8.0 at 4 °C.

Splint ligation (version 4.1, 40  µl): 1200 U T4 DNA ligase, 1 × T4 DNA Ligase Buffer, and 375 nM adapter (ExA2.1-N5/ExA2.1-20) was incubated for 1 h at 25 °C; then washed with 10 mM Tris-HCl, pH 8.0 at 4 °C.

Both version 4.0 and 4.1: DNA was eluted from the resin, and reverse cross-linking and Proteinase K treatment were performed (40 µl) containing: 30 µg Proteinase K, 25 mM Tris-HCl, pH 7.5, 2 mM EDTA, 200 mM NaCl, and 0.5% SDS incubated for 16 h at 65 °C.

The supernatant was then transferred to a new tube and purified with Agencourt AMPure magnetic beads (Beckman Coulter) following manufacturer’s instructions (1.8 × volume). The sample was eluted from the AMPure beads in 20 µl of water, and the following enzymatic steps were carried out in solution.

Second adapter ligation: ssDNA ligation (version 4.0, total reaction volume 40 µl): to the resuspended DNA was added 1200 U T4 DNA ligase, 1 × T4 DNA Ligase Buffer, 375 nM adapter (ExA2.1-N5/ExA2.1-20) and was incubated for 1 h at 25 °C.

Splint adapter ligation (version 4.1, total reaction volume 40 µl): to the resuspended DNA was added 1200 U T4 DNA ligase, 1 × T4 DNA Ligase Buffer, 375 nM adapter (ExA1-58/ExA1-SSL_N5) and was incubated for 1 h at 25 °C.

Both version 4.0 and 4.1: the ligation reaction was then purified with AMPure beads (1.8 × volume) and resuspended in 15 µl of water. The sample was then amplified via PCR. For PCR amplification (total reaction volume 40 µl); to the resuspended DNA was added 2 U Phusion Hot Start polymerase (Thermo scientific), 1 × Phusion HF Buffer (Thermo scientific), 200 µM dNTPs, 500 nM each primer (P1.3 and NexA2-iNN) and amplified for 18 cycles (20 s at 98 °C denature, 1 min at 52 °C annealing, 1 min at 72 °C extension). A quarter of the reaction was amplified for an additional six cycles (24 total) and the presence of libraries was determined by electrophoresis on a 2% agarose gel. Size selection: 200 to 500 bp PCR products were gel-purified from a 2% agarose gel using the QIAquick Gel Extraction Kit (Qiagen).

Note: ChIP-exo 4.0/4.1 incorporated a universal Read_2 adapter, with the barcode added later during PCR with long primers. Whenever long PCR primers were used in a library construction that involved lambda exonuclease digestion, the libraries suffered from low yield and high adapter dimers. We now only use full-length adapters and minimum length PCR primers.

### ChIP-exo 5.0

After immunoprecipitation, the following steps were carried out on the resin. The ChIP material on resin was washed sequentially with FA Lysis Buffer, NaCl Buffer, LiCl Buffer, and 10 mM Tris-HCl, pH 8.0 at 4 °C. For the A-tailing reaction (50 µl) containing: 15 U Klenow Fragment, -exo (NEB), 1 × NEBuffer 2, and 100 µM dATP was incubated for 30 min at 37 °C; then washed with 10 mM Tris-HCl, pH 8.0 at 4 °C. The first adapter ligation and kinase reactions (45 µl) containing: 1200 U T4 DNA ligase, 10 U T4 PNK, 1 × NEBNext Quick Ligation Buffer, and 375 nM adapter (ExA2_iNN / ExA2B) was incubated for 1 h at 25 °C; then washed with 10 mM Tris-HCl, pH 8.0 at 4 °C. The fill-in reaction (40 µl) containing: 10 U phi29 polymerase, 1 × phi29 reaction buffer, 2 × BSA, and 180 µM dNTPs was incubated for 20 min at 30 °C; then washed with 10 mM Tris-HCl, pH 8.0 at 4 °C. The λ exonuclease digestion (50 µl) containing: 10 U λ exonuclease, 1 × λ exonuclease reaction buffer, 0.1% Triton-X 100, and 5% DMSO was incubated for 30 min at 37 °C; then washed with 10 mM Tris-HCl, pH 8.0 at 4 °C.

DNA was eluted from the resin, and reverse cross-linking and Proteinase K treatment were performed (40 µl) containing: 30 µg Proteinase K, 25 mM Tris-HCl, pH 7.5, 2 mM EDTA, 200 mM NaCl, and 0.5% SDS incubated for 16 h at 65 °C. The supernatant was then transferred to a new tube and purified with Agencourt AMPure magnetic beads (Beckman Coulter) following manufacturer’s instructions (1.8 × volume).The sample was eluted from the AMPure beads in 20 µl of water, and the following enzymatic steps were carried out in solution. For second adapter ligation (total reaction volume 40 µl): to the resuspended DNA was added 1200 U T4 DNA ligase, 1 × T4 DNA Ligase Buffer, 375 nM adapter (ExA1-58/ExA1-SSL_N5) and was incubated for 1 h at 25 °C. The ligation reaction was then purified with AMPure beads (1.8 × volume) and resuspended in 15 µl of water.

The sample was then amplified via PCR. For PCR amplification (total reaction volume 40 µl); to the resuspended DNA was added 2 U Phusion Hot Start polymerase (Thermo scientific), 1 × Phusion HF Buffer (Thermo scientific), 200 µM dNTPs, 500 nM each primer (P1.3 and P2.1) and amplified for 18 cycles (20 s at 98 °C denature, 1 min at 52 °C annealing, 1 min at 72 °C extension). A quarter of the reaction was amplified for an additional six cycles (24 total) and the presence of libraries was determined by electrophoresis on a 2% agarose gel. Size selection: 200 to 500 bp PCR products were gel-purified from a 2% agarose gel using the QIAquick Gel Extraction Kit (Qiagen).

Note: we have found that using a method other than gel purification at this stage leads to an unacceptably high level of adapter dimers in the final sample. Gel purification can effectively separate the adapter dimer (150 bp fragment) and smaller fragments of the ChIP-exo library (200 bp fragment = 150 bp adapters + 50 bp insert).

### DNA sequencing

High-throughput DNA sequencing was performed with a NextSeq 500 in paired-end mode producing 2 × 40 bp reads. Sequence reads were subsequently aligned to the yeast (sacCer3) and human (hg19) genomes using bwa-mem (v0.7.9a)^24^. Aligned reads were filtered to remove non-unique alignments and PCR duplicates. PCR duplicates were defined as sequence reads possessing identical Read_1 and Read_2 sequences.

### Data availability

All sequencing files and peak files from this study are available at NCBI Gene Expression Omnibus (https://www.ncbi.nlm.nih.gov/geo/) under accession numbers GSE110681 and GSE114606. Coordinate files, script parameters, and custom code used to generate the figures for this paper can be downloaded from: https://github.com/CEGRcode/2018-Rossi_NatureCommunications.

All other data are available from the authors upon reasonable request.

## Electronic supplementary material

Supplementary Information
