# Supplementary material for: Simplified ChIP-exo assays
Source: Nat Commun. 2018 Jul 20;9:2842. doi: 10.1038/s41467-018-05265-7 (PMC6054642; doi:10.1038/s41467-018-05265-7)
Supplement: Supplementary file 1 — Supplementary Information [file 41467_2018_5265_MOESM1_ESM.pdf]

Simplified ChIP-exo assays

Rossi et al.

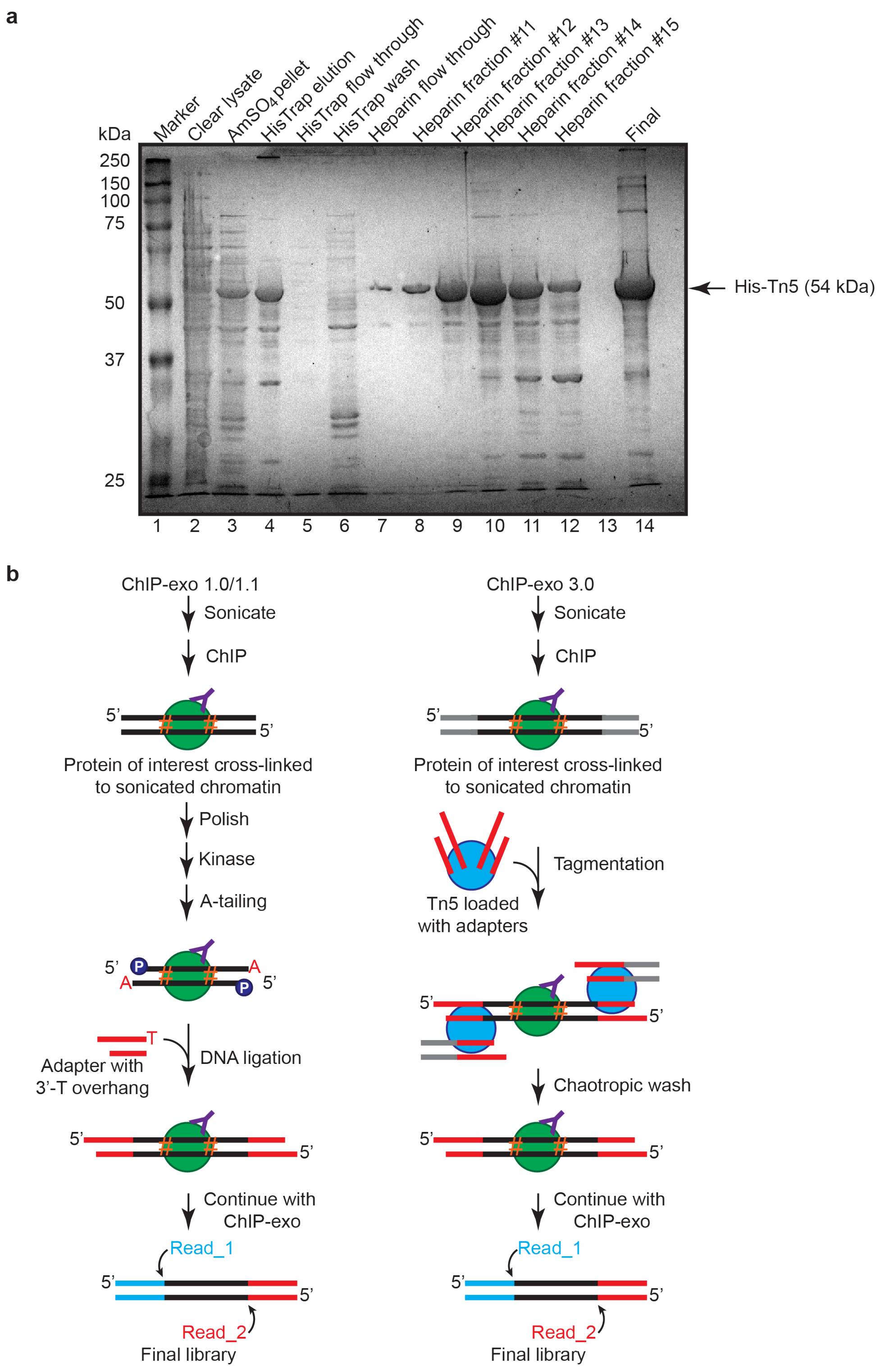

Supplementary Figure 1. Purification of hyperactive Tn5. **(a)** SDS-PAGE gel of fractions collected during Tn5 purification. Heparin fractions #12 to #14 were combined and dialyzed for the final prep. The expected size of His_6-tagged Tn5 is 54 kilodaltons. Molecular weight markers are shown in lane 1. **(b)** Schematic comparing the first steps of ChIP-exo 1.0/1.1 to ChIP-exo 3.0.

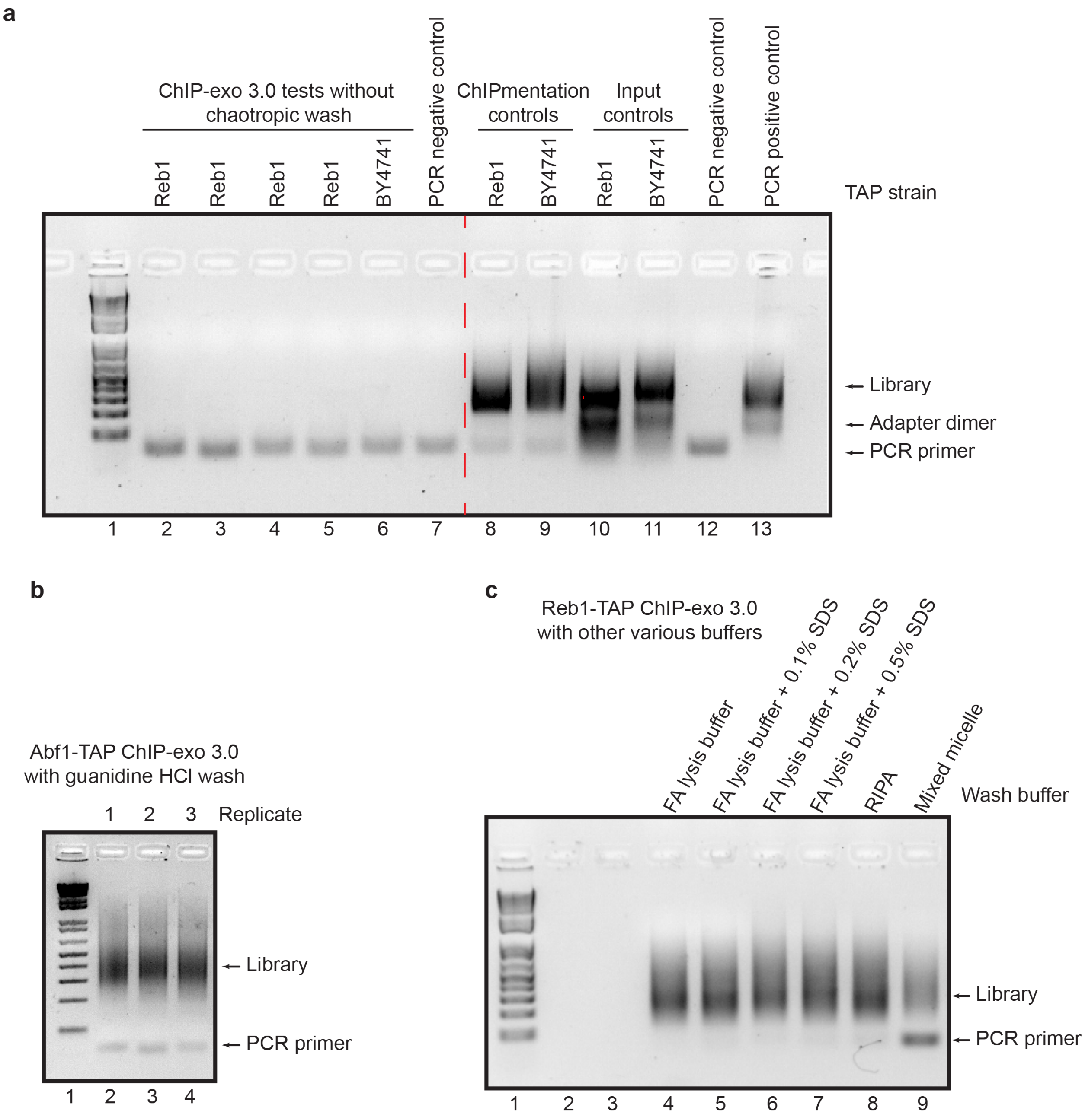

Supplementary Figure 2. ChIP-exo 3.0 requires a wash to strip away spent Tn5. 2% agarose gel of the library following 18 cycles of PCR for various ChIP-exo 3.0 libraries. **(a)** ChIP-exo 3.0 libraries did not form in the absence of a wash (lanes 2 – 6), although control samples show that the ChIP step was successful (lane 8). Robust libraries were observed when a guanidine hydrochloride buffer **(b)** or a variety of chaotropic buffers **(c)** were used to wash the sample following tagmentation.

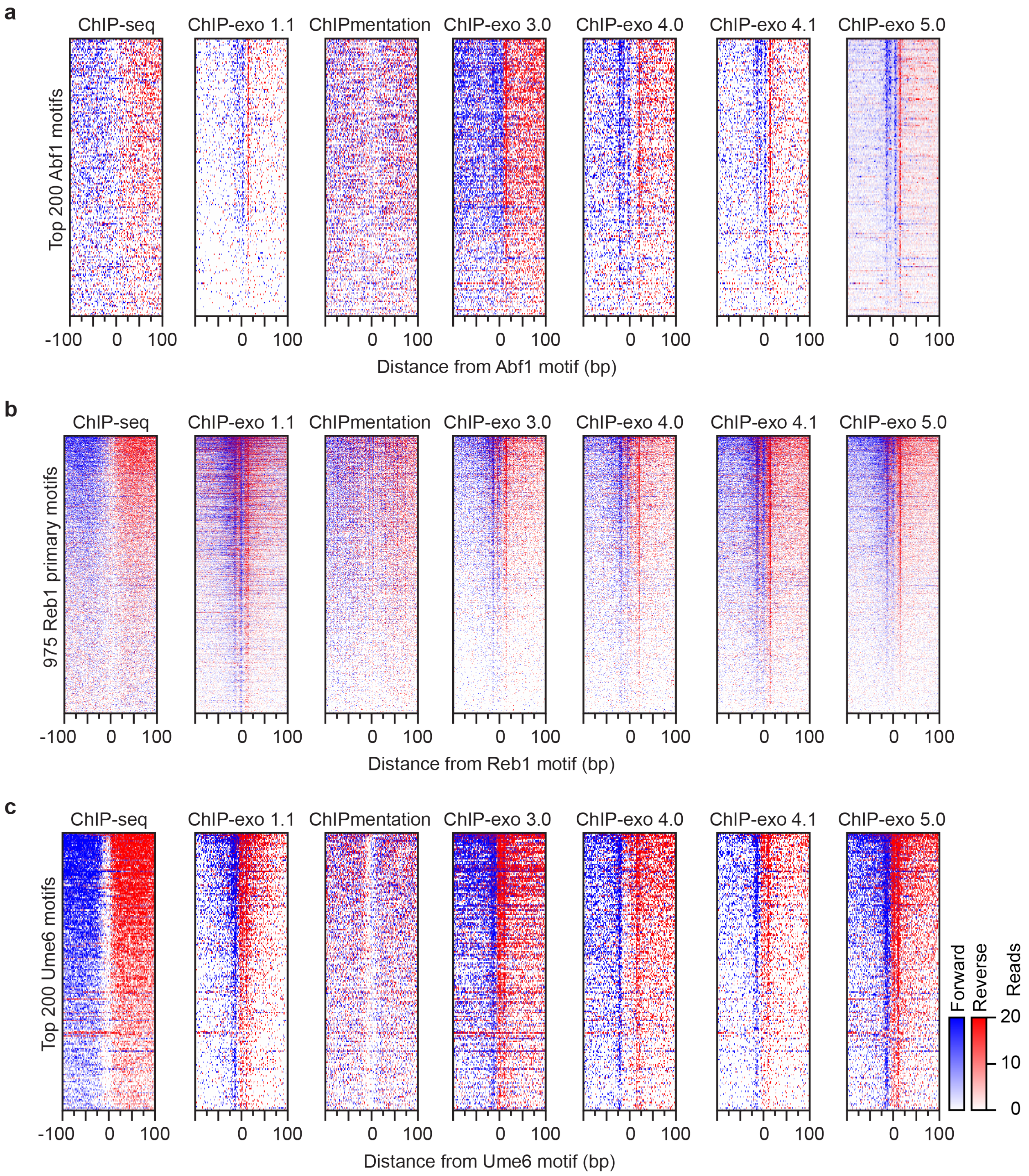

Supplementary Figure 3. Comparison of yeast transcription factors across ChIP-exo assay versions. Heatmaps of **(a)** the top 200 Abf1 motifs, **(b)** 975 Reb1 primary motifs, and **(c)** top 200 Ume6 motifs in 200 bp windows for two ChIP-seq and five ChIP-exo versions for this study. Rows are linked between factors. Each are sorted by the ChIP-exo 5.0 dataset.

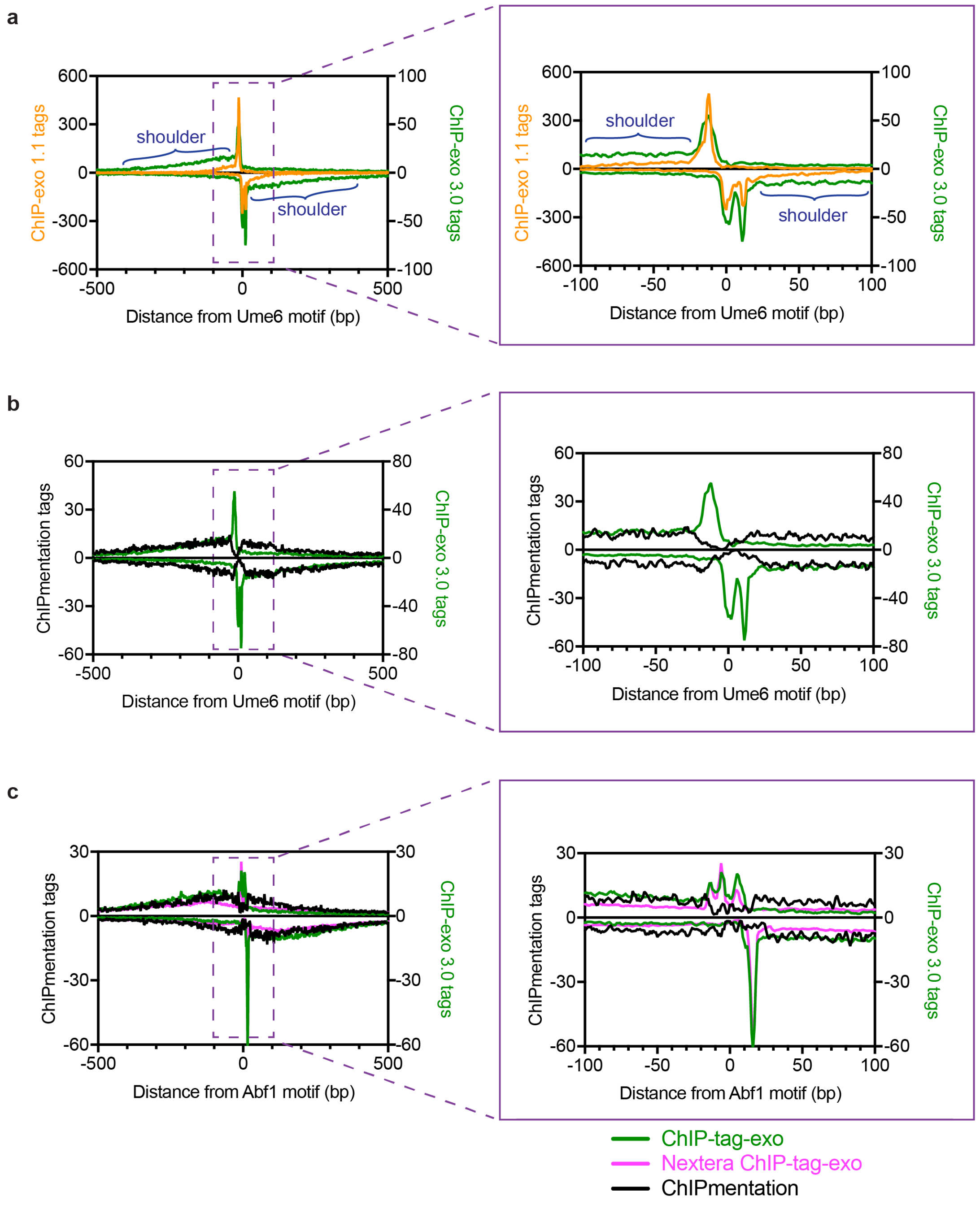

Supplementary Figure 4. Comparison to ChIP-exo 1.1 reveals shouldering observed in Tn5-based ChIP-assays (ChIP-exo 3.0 and ChIPmentation). **(a)** Strand-separated composite plot comparing ChIP-exo 1.1 and 3.0 in a 1 kb window (left) or zoomed in to 200 bp at the top 200 Ume6 motifs. Tags mapping to the strand opposite of the motif are plotted as an inverted trace (negative Y-axis). ChIP-exo 3.0 contains more tags that map hundreds of bp away from the binding site than ChIP-exo 1.1. The same high-resolution peaks are captured by both assays. **(b)** Composite plot comparing ChIPmentation and ChIP-exo 3.0 in a 1 kb window (left) or zoomed in to 200 bp at the top 200 Ume6 motifs. The shouldering seen in ChIPmentation and ChIP-exo 3.0 are very similar, but ChIPmentation lacks the high-resolution peaks seen at the binding site. **(c)** Composite plot comparing the pattern generated by the Nextera Tn5 to that of Tn5 prepared in-house as described in the Methods. The top 200 Abf1 sites are shown. Both Tn5 sources produced equivalent shouldering.

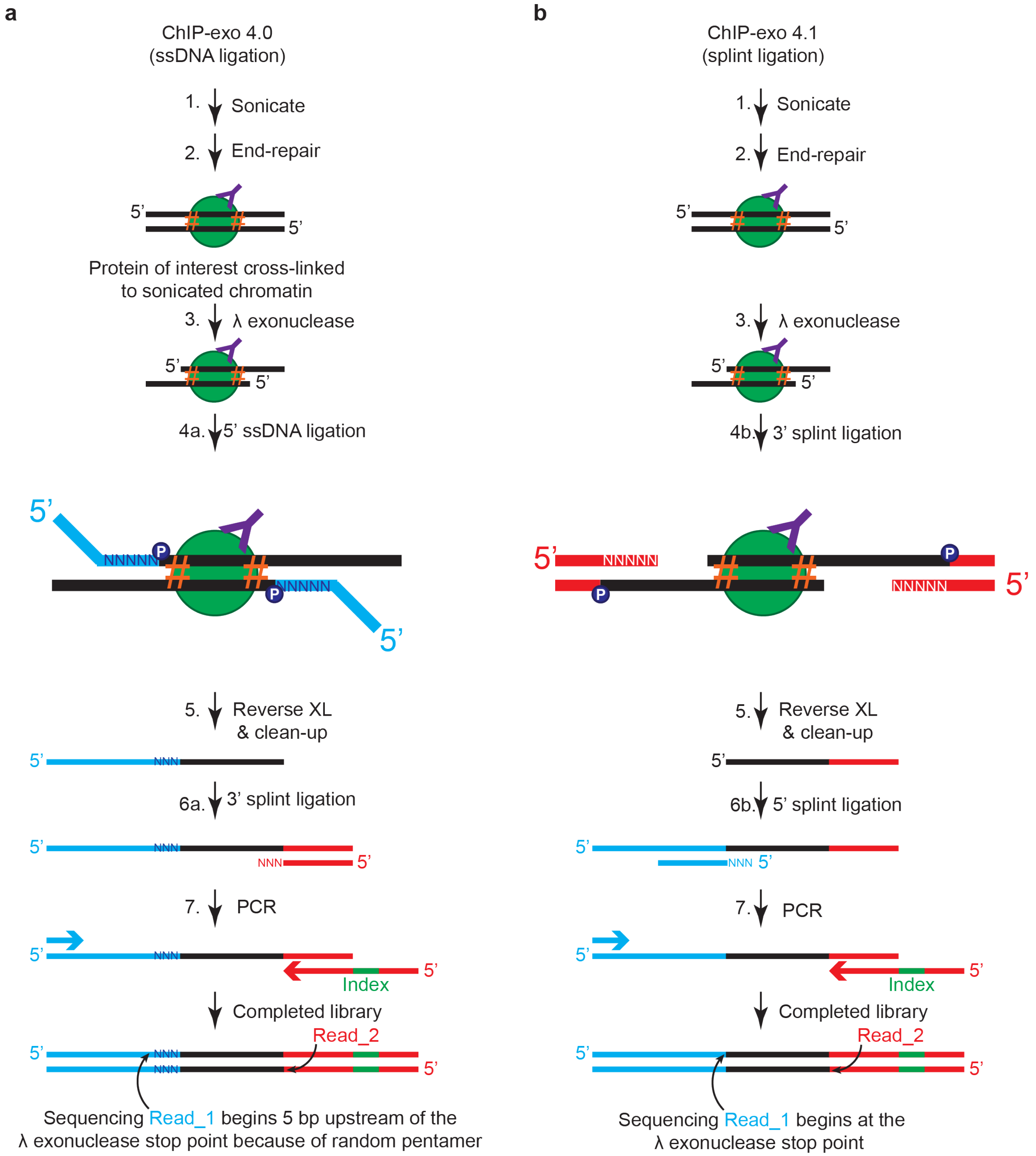

Supplementary Figure 5. ChIP-exo 4.0 and 4.1 rely on different ssDNA ligation strategies of adapters having embedded random nucleotide pentamers. Scheme for **(a)** ChIP-exo 4.0 and **(b)** ChIP-exo 4.1. These versions of ChIP-exo swap the order in which Read_1 and Read_2 adapters are ligated to the ChIP DNA, and thus involve distinct genomic substrates. In ChIP-exo 4.0, the random pentamer is incorporated immediately 5' to the exonuclease stop site, thereby shifting the peak of exonuclease stop sites by five bp when using the standard Illumina Read_1 primer. In ChIP-exo 4.1, the random pentamers anneal to the opposite strand, and thus are not incorporated into Read_1 (although are incorporated into Read_2 when conducting paired-end sequencing). The "P" represents the phosphodiester bond that is formed during ligation.

Supplementary Table 1. Oligonucleotides used in this study.

oligo name	complement oligo name	assay	step	length (nt)	sequence
NexA2	ME comp	3.0	tagmentation	39	/5Phos/AGA CGT GTG CTC TTC CGA TCA GAT GTG TAT AAG AGA CAG
ME comp	NexA2	3.0	tagmentation	19	CTG TCT CTT ATA CAC ATC T
ExA2.1-N5	ExA2.1-20	4.0<br>4.1	second ligation<br>first ligation	25	GAC GTG TGC TCT TCC GAT CTN NNN N
ExA2.1-20	ExA2.1-N5	4.0<br>4.1	second ligation<br>first ligation	20	/5Phos/AGA TCG GAA GAG CAC ACG TC
ExA1-58-N5		4.0	first ligation	63	AAT GAT ACG GCG ACC ACC GAG ATC TAC ACT CTT TCC CTA CAC GAC GCT CTT CCG ATC TNN NNN
ExA2_iNN	ExA2B	1.1<br>5.0	first ligation	70	/5Phos/CAA GCA GAA GAC GGC ATA CGA GAT XXX XXX XXX XXX GTG ACT GGA GTT CAG ACG TGT GCT CTT CCG ATC T
ExA2B	ExA2_iNN	1.1<br>5.0	first ligation	33	GAT CGG AAG AGC ACA CGT CTG AAC TCC AGT CAC
FX-15		1.1	primer extension	15	CTG GAG TTC AGA CGT
ME sequence		3.0	primer extension	19	AGA TGT GTA TAA GAG ACA G
ExA1-58	ExA1-13	1.1<br>3.0	second ligation	58	AAT GAT ACG GCG ACC ACC GAG ATC TAC ACT CTT TCC CTA CAC GAC GCT CTT CCG ATC T
	ExA1-SSL_N5	3.1<br>4.1<br>5.0	second ligation		
ExA1-13	ExA1-58	1.1<br>3.0	second ligation	13	GAT CGG AAG AGC G
ExA1-SSL_N5	ExA1-58	3.1<br>4.1<br>5.0	second ligation	19	NNN NNA GAT CGG AAG AGC G
P1.3		1.1<br>3.0, 3.1<br>4.0, 4.1<br>5.0	PCR	18	AAT GAT ACG GCG ACC ACC
NexA2-iNN		3.0<br>4.0, 4.1	PCR	69	CAA GCA GAA GAC GGC ATA CGA GAT CXX XXX XXX XXX XTG ACT GGA GTT CAG ACG TGT GCT CTT CCG ATC
P2.1		1.1<br>3.1<br>5.0	PCR	22	CAA GCA GAA GAC GGC ATA CGA G

X index sequence that varies between samples for multiplexing

N random nucleotide sequence
